# Photodissociation of aqueous $I_{3}^{-}$ observed with liquid-phase ultrafast mega-electron-volt electron diffraction

**DOI:** 10.1063/4.0000051

**Published:** 2020-12-28

**Authors:** K. Ledbetter, E. Biasin, J. P. F. Nunes, M. Centurion, K. J. Gaffney, M. Kozina, M.-F. Lin, X. Shen, J. Yang, X. J. Wang, T. J. A. Wolf, A. A. Cordones

**Affiliations:** 1 Department of Physics, Stanford University, Stanford, California 94305, USA; 2 Stanford PULSE Institute, SLAC National Accelerator Laboratory, Menlo Park, California 94025, USA; 3 Department of Physics and Astronomy, University of Nebraska-Lincoln, Lincoln, Nebraska 68588, USA; 4 SLAC National Accelerator Laboratory, Menlo Park, California 94025, USA

## Abstract

Developing femtosecond resolution methods for directly observing structural dynamics is critical to understanding complex photochemical reaction mechanisms in solution. We have used two recent developments, ultrafast mega-electron-volt electron sources and vacuum compatible sub-micron thick liquid sheet jets, to enable liquid-phase ultrafast electron diffraction (LUED). We have demonstrated the viability of LUED by investigating the photodissociation of tri-iodide initiated with a 400 nm laser pulse. This has enabled the average speed of the bond expansion to be measured during the first 750 fs of dissociation and the geminate recombination to be directly captured on the picosecond time scale.

## INTRODUCTION

Ultrafast photochemical reactions in the condensed phase are important for biological and chemical processes ranging from the first steps of vision to photocatalysis. Direct structural probes that allow us to identify atomic positions in real-time are critical to understanding the mechanisms of these photochemical reactions. Such observations of transient molecular structures have been achieved using time-resolved x-ray and electron scattering.^1–3^ Recognizing the importance of the solution environment in dictating the mechanism and dynamics of many photochemical reactions, x-ray solution scattering (XSS) at XFEL and synchrotron sources has been used to identify changes in solute geometry, bonding, and solvation, as well as molecular vibrations, on ultrafast time scales.^4–7^ Ultrafast electron diffraction (UED) presents a complementary approach to probing molecular structure in real time. Electrons are sensitive to the total Coulomb potential of the sample, making the total scattering cross section scale less strongly with the core charge than it does for x rays and allowing electron diffraction to be more sensitive to lighter elements (including hydrogen). Electron scattering in solution environments, however, has been historically challenging due to the sub-micron penetration depth of electrons in liquids, the complications multiple scattering introduces to data analysis, and the need for high vacuum. Recently, the development of mega-electron-volt (MeV) UED and gas-accelerated liquid sheet jets^8^ that produce sub-micron, in-vacuum liquid samples made possible the development of a liquid phase UED (LUED) instrument^9^ with sub-200 femtosecond time resolution. Here, we pioneer the use of LUED to study the reaction dynamics of a molecule in solution.

The ability of LUED to directly observe solute structural dynamics is demonstrated on a well-characterized reaction, the photodissociation of tri-iodide ($I_{3}^{-}$) in water by 400 nm excitation. $I_{3}^{-}$ photodissociation has been extensively studied over several decades in both the gas and solution phase via a variety of methods. Gas-phase photofragment mass spectrometry experiments determined photon energy-dependent branching ratios between three-body dissociation ($I_{3}^{-}$ → I^−^ + 2I) and two types of two-body dissociation, $I_{3}^{-}$ → $I_{2}^{-}$ + I and $I_{3}^{-}$ → I_2_ + I^−^.^10^ In solution, the latter type of two-body dissociation is not observed,^10–12^ suggesting a strong effect of the solvent on the escaping fragments. Solution-phase photon energy-dependent studies determined that for excitation at 400 nm in ethanol, only the $I_{2}^{-}$ + I two-body dissociation occurs, while higher photon energy excitation results in both two- and three-body dissociation.^13^ The single dissociation channel at 400 nm was also confirmed in water via picosecond time-resolved XSS measurements of the $I_{2}^{-}$ product formed following dissociation.^14^ XSS was also used to establish the ground-state structure of the $I_{3}^{-}$ anion in polar solvents.^15^ This structural study of Kim *et al.*^15^ provides further support that $I_{3}^{-}$ adopts an asymmetric structure in H-bonding solvents, with charge concentrated on one terminal I atom which is further apart from the central I than the opposite terminal atom.^16–19^ In water, the structure of $I_{3}^{-}$ was measured via XSS measurements to be substantially bent as well as asymmetric.^15^

Many ultrafast optical spectroscopy studies have focused on the vibrational and rotational dynamics of the product species, as the nascent $I_{2}^{-}$ can be tracked spectroscopically.^11–13,20^ Femtosecond-resolution scattering measurements such as LUED have the potential to offer complementary information about the bond breaking process in real time, since electron scattering directly encodes interatomic distances, in contrast to optical measurements, where electronic and nuclear dynamics can prove difficult to distinguish.

Here, we present LUED measurements of $I_{3}^{-}$ in water excited with 400 nm light on two time scales. First, the population of product species $I_{2}^{-}$ + I is tracked over a range of 10 ps via comparison to Molecular Dynamics (MD)-simulated difference scattering, and a signature attributable to geminate recombination is observed on the time scale of ∼1 ps. Then, with femtosecond time resolution in the sub-picosecond regime, the effect of the increasing I–I distance during photodissociation is directly observed in the difference scattering, and an estimated average dissociation speed is extracted.

## RESULTS AND DISCUSSION

We have tracked the structural dynamics of $I_{3}^{-}$ during photodissociation using optical pump-MeV electron probe experiments which are fully described in the Methods section. Briefly, a sub-200 nm sheet jet of aqueous $I_{3}^{-}$ solution (prepared from 130 mM I_2_ and excess KI) was pumped with a 400 nm optical laser pulse to initiate photodissociation, and the scattered 3.7 MeV electron beam probe was detected downstream. Azimuthally integrated scattering patterns from negative pump-probe time delays were subtracted from the entire dataset to construct difference scattering curves with magnitudes less than 0.5% of the total scattering (supplementary material Fig. S1). All analysis is done on difference scattering curves, as the concentration of solute is not sufficient to extract static structures, consistent with x-ray studies.^15^

While the LUED measurements remain the focus of this work, optical transient absorption in the UV-Visible regime was also conducted to investigate if other photochemical pathways beyond the two-body dissociation observed previously^13,14^ are possible under the high laser power conditions used for the LUED experiments (40 and 60 *μ*J with a laser spot size of 160 × 270 *μ*m). To quantify the expected yield of other pathways, optical transient absorption measurements were conducted with the same maximum optical intensity (supplementary material Sec. II). These show that approximately 85% of the photoproducts are consistent with $I_{2}^{-}$ + I, with the remainder resulting from photoionization of $I_{3}^{-}$ (supplementary material Fig. S3). The secondary ionization pathway does not create sufficiently large changes in the pair distribution function to be detected with the achieved signal to noise of our LUED measurements (supplementary material Fig. S4). Therefore, the LUED data were analyzed only with respect to the dominant two-body dissociation pathway, $I_{3}^{-}$ → $I_{2}^{-}$ + I.

In the following, we present LUED data collected in two modes: a high electron bunch charge mode with ∼750 fs (FWHM) time resolution, used to probe dynamics up to 10 ps after the laser pulse, and a low electron bunch charge mode with 180 fs (FWHM) time resolution, used to probe sub-picosecond dynamics. We first show that the population of $I_{2}^{-}$ + I product fragments can be tracked on the longer time scale by comparison with MD simulations, revealing a picosecond decay of product population which is assigned to geminate recombination. Then, we show that dissociation can be observed with ultrafast time resolution within the first 750 fs.

### Picosecond population dynamics of $I_{2}^{-}$ and I product fragments through comparison of LUED data and MD simulations

**FIG. 1.**
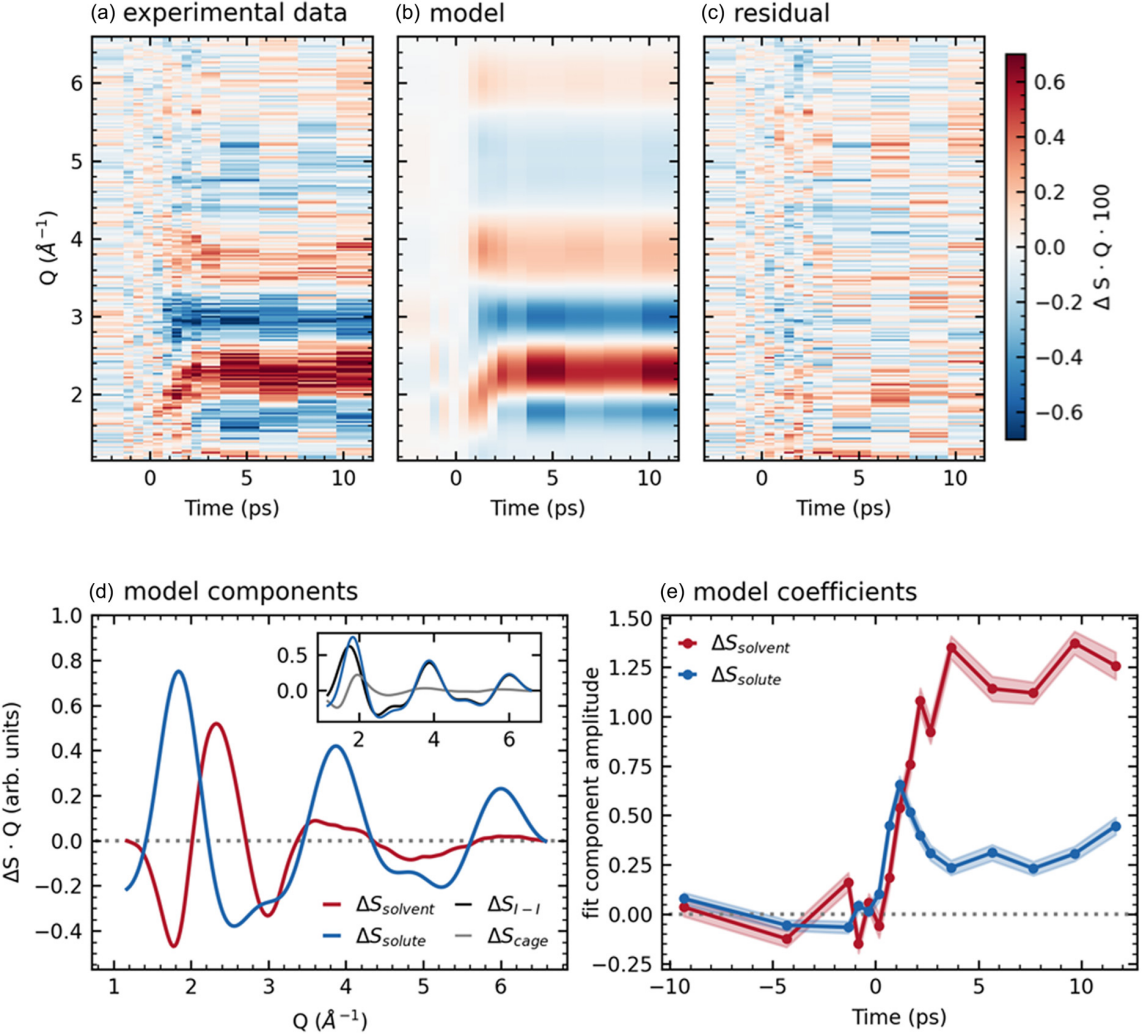
Picosecond time scale difference scattering data, modeled as a linear combination of solvent heating and solute-associated contributions. (a) Difference scattering data ΔS·Q. (b) Modeled ΔS·Q, obtained by multiplying the curves in (d) and (e). (c) Residuals of the fit. (d) The two components of the model in *Q*-space: the measured $\Delta S_{\mathrm{solvent}}$ (red) and the simulated $\Delta S_{\mathrm{solute}}$ (blue). Inset: $\Delta S_{\mathrm{solute}}$ (blue) consists of contributions from solute–solute atom pairs ($\Delta S_{I-I}$, black) and solute–solvent pairs ($\Delta S_{\mathrm{cage}}$, gray). (e) Time traces for the two components obtained from fitting data at each time delay to a weighted sum of the components.

Picosecond-resolution difference scattering patterns obtained over a 10 ps range using the high electron bunch charge mode of the UED instrument are shown in Fig. 1. The azimuthally averaged difference scattering ΔS, multiplied by the momentum transfer *Q* to make signal at higher momentum transfer more visible, is shown in Fig. 1(a). The low signal-to-noise in the data above *Q *=* *6 Å^–1^, combined with the strong overlap between the difference scattering from the changes in I–I bond length and changes in the water structure factor due to solvent thermalization, preclude the robust sine transform of the difference signal into real space. Instead, we focus our analysis on dissociation-associated features in the *Q*-space difference scattering ΔS·Q.

As established for the analysis of time-resolved XSS,^21–28^ ΔS·Q comprises a solute-associated signal, arising from photoinduced changes of solute–solute and solute–solvent atom pair distances, and a bulk solvent signal, arising from heat deposition following solute relaxation. Therefore, to characterize the behavior of the dissociation products, we model the experimental ΔS·Q as a linear combination of solute-associated [$\Delta S_{\mathrm{solute}}(Q)$] and bulk solvent [$\Delta S_{\mathrm{solvent}}(Q)$] signals. $\Delta S_{\mathrm{solute}}$ comprises the difference scattering due to changes in both the intramolecular structure (solute–solute) and the solvation cage structure (solute–solvent), and was derived from classical MD simulations as described below. $\Delta S_{\mathrm{solvent}}$, which arises from heating of the bulk solvent as energy is deposited in the system, was extracted from LUED data of vibrationally excited pure water. Extensive characterization of laser-induced solvent heating using time-resolved XSS has previously demonstrated that its shape is independent of the heating mechanism and does not appreciably evolve in time, even on ultrafast time scales.^24–27^ $\Delta S_{\mathrm{solvent}}$ was extracted from LUED data of pure water heated via excitation with 3 *μ*m light, averaged for pump-probe delays between 5 and 10 ps [Fig. 1(d), red line]. The independently measured $\Delta S_{\mathrm{solvent}}$ is consistent with XSS measurements of water heating^24,29^ as well as molecular dynamics simulations,^9^ as shown in supplementary material Fig. S6.

We simulated $\Delta S_{\mathrm{solute}}$ using classical MD (the details of which are given in the Methods). $\Delta S_{\mathrm{solute}}$ contains information about the structural changes of the solute and the relative reorganization of the solvent shells surrounding the solute fragments. The system before dissociation was modeled as one $I_{3}^{-}$ anion, one K^+^ cation, and 1350 water molecules. The system after dissociation consisted of one $I_{2}^{-}$ anion, one neutral I atom, one K^+^ cation, and 1350 water molecules. The fixed geometries (bond lengths and angles) and partial charges of the anions were taken from Ref. 15 and are shown in supplementary material Table S1. A simulated difference scattering curve $\Delta S_{\mathrm{solute}}$, constructed as the difference between the before- and after-dissociation trajectories, was obtained following the method of Ref. 30 with atomic form factors from the ELSEPA program^31^ and is shown in blue in Fig. 1(d). This curve models the expected signal after fragment separation. By separating the MD simulation into solute–solute and solute–solvent components [Fig. 1(d) inset], we find that $\Delta S_{\mathrm{solute}}$ is dominated by the change in iodine–iodine atom pair distances, with the lighter elements of the solvent cage contributing only slightly to the total signal. Coherent structural vibrations of $I_{3}^{-}$ or of the $I_{2}^{-}$ fragment^20^ are not included in our MD simulation, as the time resolution of this dataset is not sufficient to observe these vibrations.

In the first picosecond, as reported in the high time-resolution dataset below, bond breakage is directly visible as a change in the shape of ΔS·Q. After this point, however, collisions with solvent begin to broaden the distribution of distances between the I and $I_{2}^{-}$ fragments. Previous MD simulations of $I_{3}^{-}$ dissociation dynamics^32^ suggest that by the first picosecond following excitation, the distance between product fragments is broadly distributed, with a width of ≈ 5 Å. Therefore, we expect the picosecond-scale difference scattering data reported in Fig. 1(a) to reflect the average of many discrete product distances even at the earliest time points available at this resolution. Although our MD simulations allow the fragment separation to randomly diffuse over the full simulation box, we find that the simulated difference signal is still a valid measure of the expected sub-10 Å product distributions in the first few picoseconds. This is demonstrated in supplementary material Fig. S7, which compares the MD result to the average of simulations with discrete fragment separation distances of 1–6 Å and 5–10 Å (these simulations only include the iodine atoms; difference scattering curves were obtained via the Debye equation). The difference scattering signal for products that remain in close range (1–6 Å), as well as that for products that are further separated (5–10 Å), both match sufficiently with the MD simulation. The MD-derived signal is therefore used as a measure of the population of dissociated molecules, regardless of the exact I–$I_{2}^{-}$ distance, for the time delays considered in Fig. 1.

Each pump-probe delay in the experimental ΔS·Q was fit to a linear combination of the MD-simulated $\Delta S_{\mathrm{solute}}$ and the experimentally measured $\Delta S_{\mathrm{solvent}}$ in the range *Q *=* *1.15–6.60 Å^– 1^ (full data range shown in supplementary material Fig. S8). The results of this fit are shown in Fig. 1(e). The coefficients of the two components are plotted as a function of time delay in panel (e), with error bars from the square root of diagonal covariance matrix elements of the least squares fit. $\Delta S_{\mathrm{solvent}}(Q,t)$ rises to a plateau in the first 4 ps, consistent with the rapid growth of heat-associated signal observed in XSS experiments in aqueous solution.^26,27^ $\Delta S_{\mathrm{solute}}(Q,t)$, on the other hand, rises to a peak within 1 ps, and then rapidly decays to a lower plateau. This decay in $\Delta S_{\mathrm{solute}}(Q,t)$ indicates the decreasing population of the dissociated product fragments and is interpreted as a signature of geminate recombination.

The peaked behavior of $\Delta S_{\mathrm{solute}}(Q,t)$ is consistent with rapid separation of the $I_{2}^{-}$ and I fragments (as also reported in the high time-resolution dataset below), until they collide with the solvent cage. The nature of these collisions dictates the branching between fragments that recombine and those that remain dissociated. The final plateau of the solute component represents those fragments which escape the solvent cage and remain dissociated. Thus, the time-dependence of $\Delta S_{\mathrm{solute}}(Q,t)$ contains information about the time scale of geminate recombination and the probability of solvent cage escape.

**FIG. 2.**
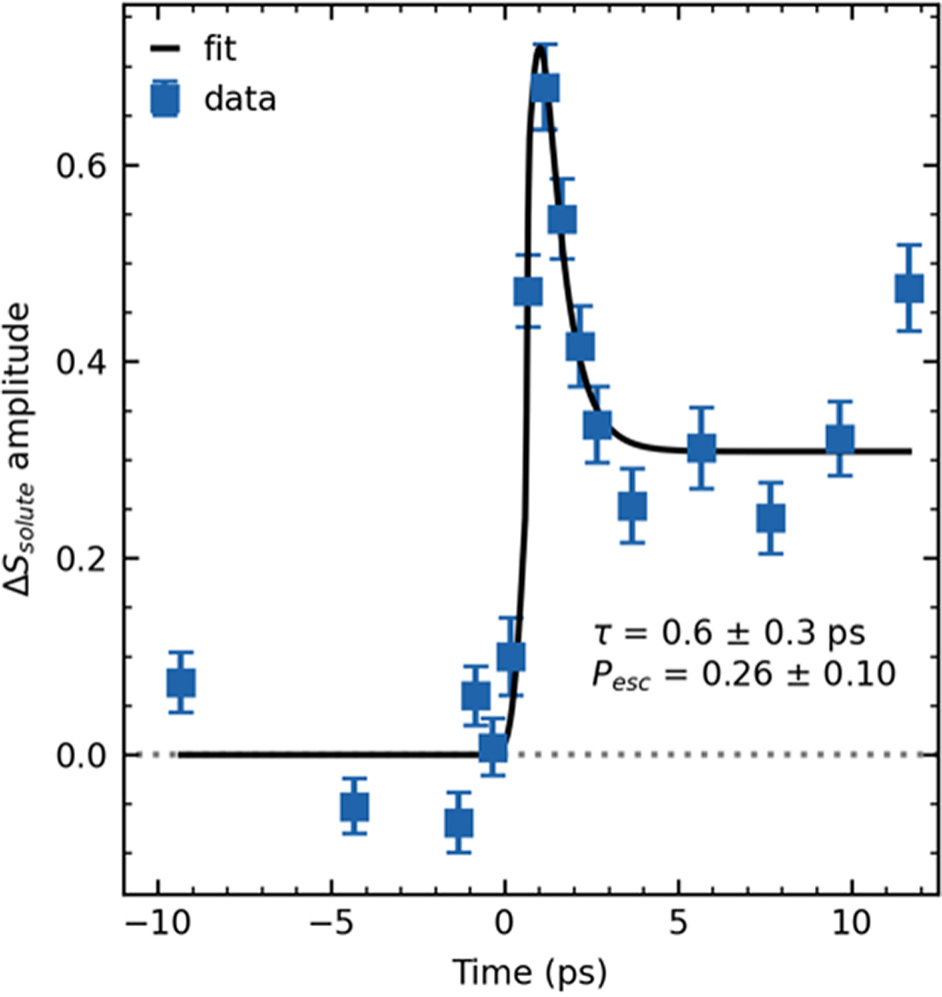
Time dependence of the magnitude of the product signal component $\Delta S_{\mathrm{solute}}$ (blue) and fit to Eq. (1) (black), modeling geminate recombination, with instrument time resolution set to 750 fs FWHM.

In Fig. 2, the time trace of the solute contribution was fit to the following function, where *A_rec_* represents the population that recombines and *A_esc_* represents the population that escapes:

$$f(t)=\text{exp}\;\left(\frac{-t^{2}}{2\sigma^{2}}\right)*\left\{\begin{array}{cc} 0 & t\leq t_{0}\\ A_{\mathrm{rec}}\;\text{exp}\;\left(\frac{-(t-t_{0})}{\tau_{\mathrm{rec}}}\right)+A_{\mathrm{esc}} & t>t_{0} \end{array}\right\}.$$ (1)

This function represents an instantaneous (within the picosecond resolution of the dataset) jump of signal at *t* = *t*_0_ to a value of *A_rec_*+*A_esc_*, which subsequently decays to a value *A_esc_* with a time constant *τ_rec_*. The signal is convolved with a Gaussian instrument response function (IRF) with a full width at half-maximum (FWHM) of 0.75 ps (FWHM = 2.355σ). The escape probability is given by the ratio of final to initial signal level: $P_{\mathrm{esc}}=A_{\mathrm{esc}}/(A_{\mathrm{rec}}+A_{\mathrm{esc}})$, while the time scale of geminate recombination is given by *τ_rec_*. This fitting function does not consider secondary recombination time scales reported previously, on the order of 40 ps,^33^ which fall outside of our measurement window. The fit results are shown in Fig. 2, yielding a recombination time scale *τ_rec_* of 0.6 ± 0.3 ps and an escape probability *P_esc_* of 0.26 ± 0.1. These fit results are similar to the transient absorption measurements of Gershgoren *et al.*,^33^ which found a partial recovery of the $I_{3}^{-}$ spectrum on a time scale of 1.3 ps in water, corresponding to fast recombination, with $P_{\mathrm{esc}}<$ 33%. Overall, our picosecond-time scale LUED measurements provide a quantitative measure of the dissociated population dynamics through comparison with MD-simulated difference scattering.

### Ultrafast photodissociation of $I_{3}^{-}$: Sensitivity of LUED to bond distance

**FIG. 3.**
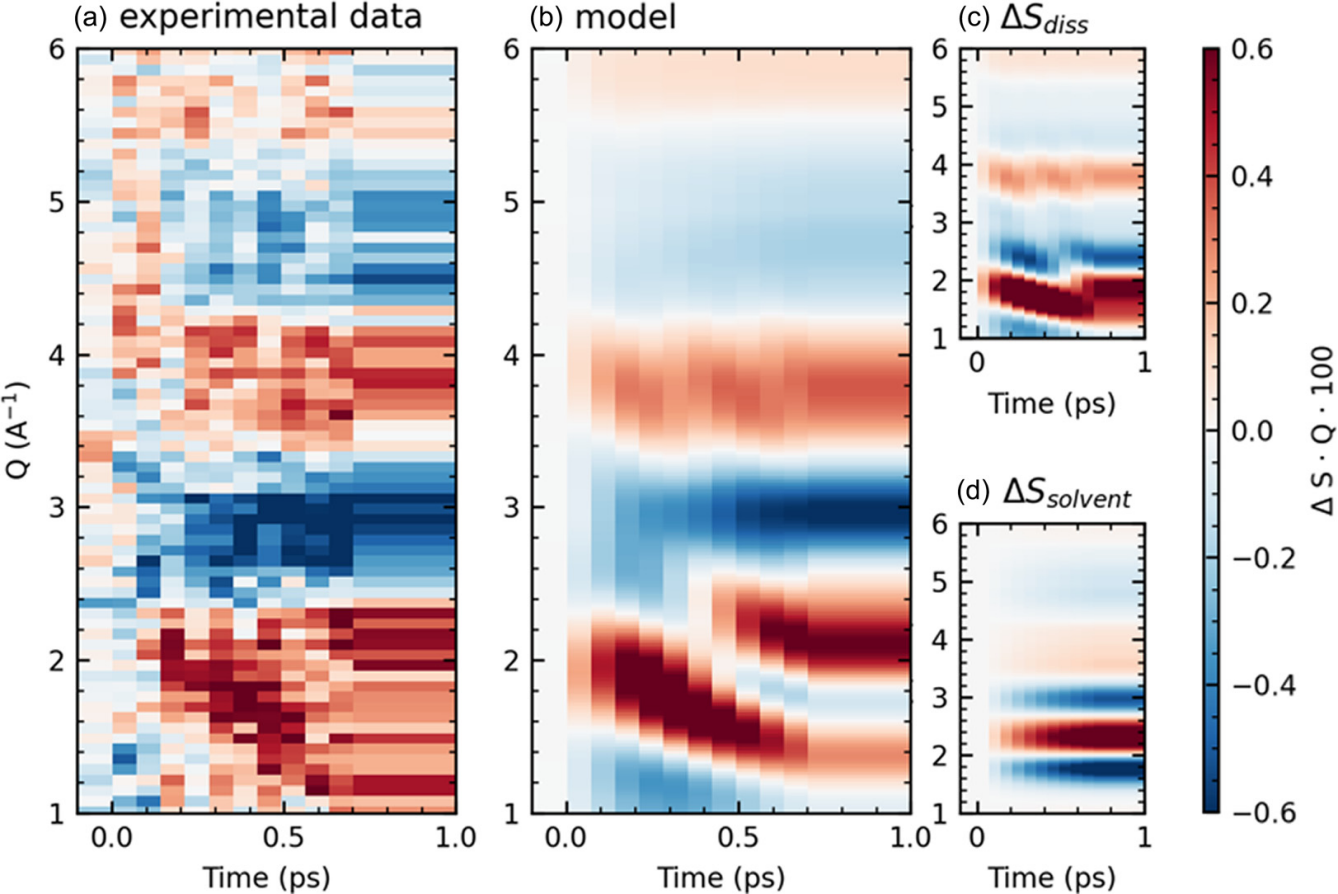
Experimental (a) and simulated (b) difference scattering patterns. The model in (b) is the sum of a three-atom model of the solute dissociation (c) as described in the text with optimum parameters *v *=* *5.8 Å/ps (optimum *t*_0_ set to *t *=* *0), and a water heat component $\Delta S_{\mathrm{solvent}}(Q,t)$ (d) obtained by multiplying $\Delta S_{\mathrm{solvent}}$ by a smooth fit to the measured solvent heating background, as described in the text.

High time resolution difference scattering patterns were obtained over a 2 ps range using the low electron bunch charge mode of the UED instrument. Due to the weaker signal of the small electron bunch, long data acquisition times are required, and fine time delay steps were only acquired for the first 750 fs following excitation. ΔS·Q from this measurement is shown in Fig. 3(a). In this difference signal, the positive feature at *Q *=* *1–2 Å^–1^, which starts at higher *Q* and moves to lower *Q* values as a function of time, provides a clear signature of solute dissociation in agreement with previously published XSS signals.^34–36^ As atoms move apart, higher frequency modulations appear in the scattering pattern, leading to characteristic “stripes” in the difference scattering as peaks and nodes of the new frequencies pass through those present in the initial state. In the higher Q range > 1.5 Å^–1^, contributions of the characteristic signal arising from a temperature increase in bulk water ($\Delta S_{\mathrm{solvent}}$) are also observed.

Figure 3(b) shows that a simulated signal constructed from a linear combination of a solute dissociation signal [$\Delta S_{\mathrm{diss}}$, shown in Fig. 3(c)] and the measured bulk solvent heating signal $\Delta S_{\mathrm{solvent}}$ [Fig. 3(d)] capture the main features of the data. The simulated $\Delta S_{\mathrm{diss}}(Q,t)$ in panel (c) is derived from a simple three-atom model in which the scattering is calculated as one iodine atom is moved away from the other two. Calculated scattering curves are obtained under the independent atom model (IAM), using the Debye equation with atomic form factors from the ELSEPA program.^31^ The changing I–I distance is put into the relevant frame of delay time by assuming a fixed average dissociation speed of 5.8 ± 0.3 Å/ps. This approximation of the dissociation speed was extracted from the experimental data as described below. $\Delta S_{\mathrm{solvent}}(Q,t)$ in panel (d) is derived from least squares fitting of the measured ΔS·Q at each time-point to $c(t)\cdot \Delta S_{\mathrm{solvent}}(Q)$ to obtain the contribution of the heating component to the data (described below and in supplementary material Fig. S10). The combination of the two model components, shown in panel (b), yields a good qualitative match to the experimental ΔS·Q, demonstrating the direct observation of bond dissociation of a solvated molecule using LUED. The limited signal-to-noise and time window of this proof of principle experiment do not warrant the inclusion of additional photochemical phenomena, such as the coherent motion of the nascent $I_{2}^{-}$ fragment observed in optical studies.^20^ It also justifies the use of the overly simplistic three-atom dissociation model that neglects solute–solvent contributions to the difference scattering signal (this is further justified in supplementary material Sec. IV, Fig. S9).

The speed of dissociation is encoded in the rate at which the positive peak in the area between 1 and 2 Å^–1^ shifts to lower *Q* values. Due to the low signal-to-noise of the data, solute structural parameters cannot be robustly extracted by fitting a model comprising both solute and solvent signals to the full ΔS·Q. To extract the speed, the data are instead treated in two steps: first $\Delta S_{\mathrm{solvent}}(Q,t)$ is subtracted from the data, then the remaining signal is fit and compared to the three-atom dissociation model.

Heat transfer to the bulk solvent is found to occur rapidly, and is visible even within the first 750 fs, consistent with XSS measurements of aqueous systems.^28^ This signal [red trace, Fig. 1(d)] overlaps with the low-Q “stripe” feature of interest and must be removed prior to comparing the data to the $\Delta S_{\mathrm{diss}}$. Therefore, the difference scattering at each time delay is fit to $c(t)\cdot \Delta S_{\mathrm{solvent}}(Q)$ in the region Q = 2.5–4.25 Å^–1^. The bounds of the fit region were chosen to include the area of the largest magnitude of the heating signal, while avoiding overlap with the low-*Q* solute signal of interest. Varying the lower bound of the fit region influences the magnitude of the heating signal fit (*c*(*t*)), but the dissociation speed extracted from this analysis is affected by less than the reported 1*σ* error (see supplementary material Fig. S10). *c*(*t*) is plotted in supplementary material Fig. S10 and can be described by an exponential growth. The 2-dimensional $\Delta S_{\mathrm{solvent}}$ in Fig. 3(d) is constructed by multiplying $\Delta S_{\mathrm{solvent}}(Q)$ by the exponential growth describing *c*(*t*).

**FIG. 4.**
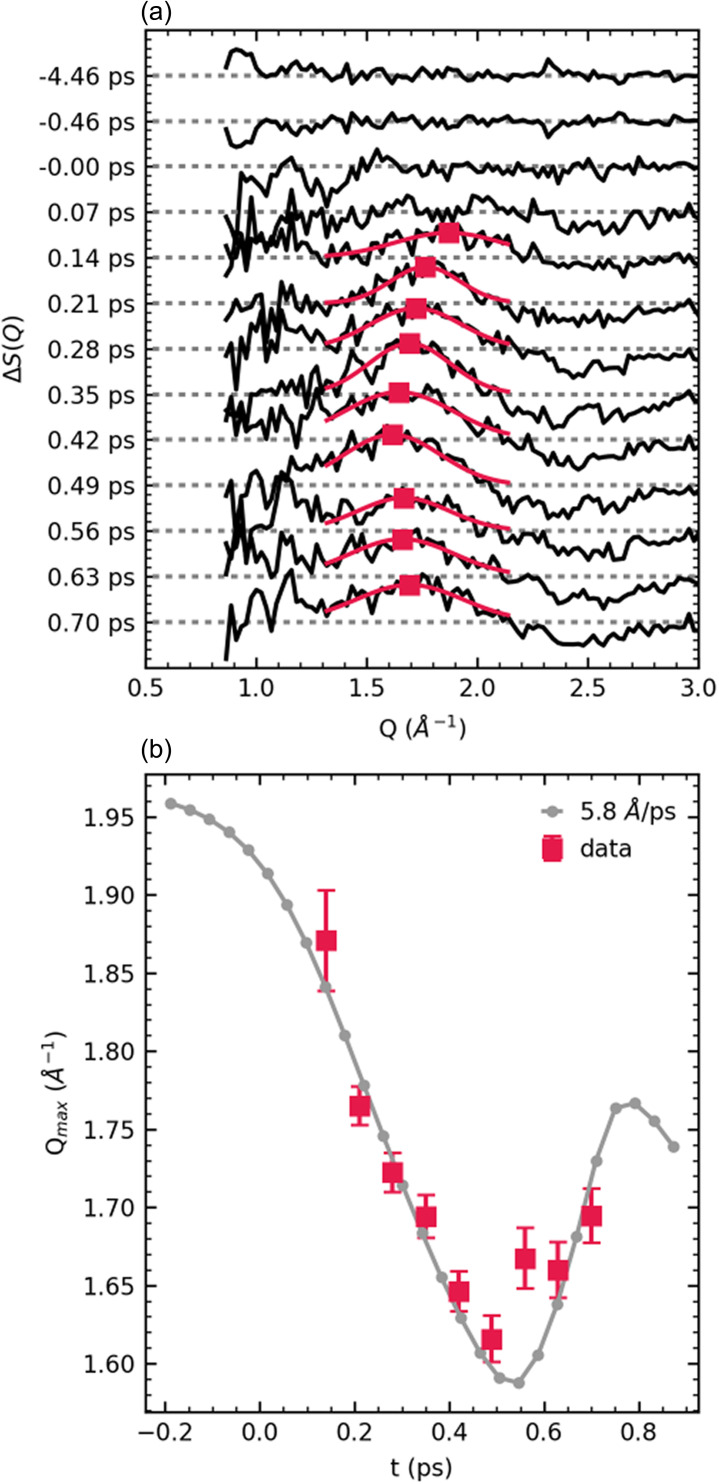
(a) Difference scattering data (black) at various time delays after solvent heat signal subtraction, as described in the text. The *Q *=* *1.30–2.15 Å^–1^ region is fit to a Gaussian function (red) at each time delay, and the peak position (red squares) is plotted in panel (b) as a function of time. These peak positions are fit to a three-atom dissociation model, as described in the text. The result of this model with best-fit dissociation speed *v *=* *5.8 Å/ps is plotted in panel (b) (gray). The time axes have been shifted to place the best-fit *t*_0_ at *t *=* *0; the time resolution is fixed to 180 fs FWHM.

Figure 4(a) shows the difference scattering data in the region of interest after subtraction of $c(t)\cdot \Delta S_{\mathrm{solvent}}(Q)$. This experimental data were compared to the simple three-atom model $\Delta S_{\mathrm{diss}}$ to assign the average dissociation speed. In $\Delta S_{\mathrm{diss}}$, the ground state $I_{3}^{-}$ geometry of Ref. 15 was altered by moving one of the terminal iodine atoms away from the other two at a constant speed along its bond axis, while the other terminal iodine moved at the same speed to adjust the remaining bond length to 3.43 Å (the ground-state bond length of the final $I_{2}^{-}$ fragment). Due to the asymmetric structure of the $I_{3}^{-}$ ion in water solution,^15–18^ there are two nonequivalent bonds that could be broken. *Ab initio* calculations^37^ suggest that the final products observed in solution, $I_{2}^{-}$ and I, are the most stable products only along the reaction coordinate which breaks the shorter I–I bond. We therefore only show models of short bond breakage, though a model breaking the longer bond is shown in supplementary material Fig. S11 for comparison, and the best-fit dissociation speed is unchanged.

Simulated scattering patterns as a function of time delay (as opposed to I–I distance) were developed using average dissociation speed *v* and time zero *t*_0_ as freely varying fit parameters. The use of a constant average speed is an approximation, as the atoms are accelerating and decelerating within the time window of the measurement. However, time resolution and signal levels are insufficient to map acceleration. Simulated trajectories of the I atoms were converted to difference scattering via the Debye equation. The resulting simulated $\Delta S_{\mathrm{diss}}(Q,t)$ was broadened by the temporal resolution of 180 fs FWHM, and the peak position of the simulated low-*Q* positive “stripe” feature was obtained and compared to the experiment. The peak positions were fit to a Gaussian function in the range *Q *=* *1.30–2.15 Å^–1^ (for both the simulated $\Delta S_{\mathrm{diss}}$ and measured ΔS·Q); fits to the experimental data are shown by the red lines in Fig. 4(a). These peak positions as a function of time are shown in panel (b) and display an initial decrease toward lower *Q*, followed by an increase, consistent with the three-atom model; as higher spatial frequencies appear in the scattering pattern as a function of time, the result in a narrow *Q* range is a peak moving toward lower values, until the next peak of the pattern appears and moves through the same region. A maximum likelihood estimate (MLE) framework was used to assess the quality of the fit for a range of (*v*, *t*_0_) pairs as shown in supplementary material Fig. S11.

From this analysis, the average dissociation speed is estimated to be 5.8 ± 0.3 Å/ps, and this best-fit simulated curve is plotted in gray in Fig. 4(b); the time axes in Fig. 4 have been shifted to place the fitted *t*_0_ at *t *=* *0. The model of the total ΔS·Q based on this dissociation speed is shown in Fig. 3(c).

The agreement between the simple dissociation model and the data strongly suggests that $I_{3}^{-}$ → $I_{2}^{-}$ + I is being directly observed. However, as discussed above, the high laser power of the experiment (180 mJ/cm^2^) enables secondary photochemical pathways. Photoionization is the dominant secondary pathway based on Uv-Vis transient absorption results (supplementary material Sec. II); this generates the I_3_ radical species, which has been observed in the gas phase to be marginally stable.^38^ The quantum chemistry predicted difference in bond length between $I_{3}^{-}$ and I_3_ would appear to be too small to generate an appreciable difference signal for this minority channel (supplementary material Fig. S4), though secondary dissociation of I_3_ could also be occurring in the experiment. Even in the presence of this secondary source of dissociation dynamics, the speed extracted from the dissociation signature can be qualitatively compared to simulations and previous measurements, and can be used as a proof of principle for the type of information available in LUED.

The speed of dissociation in $I_{3}^{-}$ in ethanol has been estimated by the MD simulations of Benjamin *et al.*,^32^ which suggest motion on the order of 2–4 Å/ps for the first 2 ps. Due to the complexity of quantum dynamics studies in solution, calculated estimates of dissociation speeds from quantum mechanical models are more readily available in the gas phase and are several times higher, in the 15–25 Å/ps range.^35,39^ The fact that the speed extracted from our LUED measurement is so much lower than gas-phase estimates points to a strong effect of the solvent on the potential governing the dissociative motion. Studies comparing the bond fission time scales of small organic molecules in gas and cyclohexane via UV/Vis and IR transient absorption have determined that the potentials involved in the initial bond breaking are not strongly perturbed by the solvent.^40^ However, this result is not necessarily transferable to polar solvents like water,^40,41^ and indeed our measurement implies that in this case, there is substantial interaction with and transfer of energy to the solvent which would preclude using gas-phase potentials to model the system, even at early times. The interatomic distances probed by LUED are not readily available from optical measurements, making the measurement complementary to more established methods. Direct observation of dissociation speeds by scattering experiments has also previously been demonstrated with both x rays and electrons in the gas phase^36,42,43^ and with x rays in solution.^44^ As LUED develops, direct comparisons between gas- and liquid-phase experiments could shed light on the effect of solvent on the potentials governing dissociative motion.

## CLOSING REMARKS

We have demonstrated the use of liquid-phase ultrafast electron diffraction to observe photochemical reaction dynamics of a solvated molecule, aqueous $I_{3}^{-}$, for the first time. Difference scattering directly encodes photo-dissociative motion, and comparison of difference scattering to MD simulations suggests rapid geminate recombination in the first picosecond after dissociation. While previously the high level of structural sensitivity and time resolution available to MeV-UED had been restricted to solid and gaseous samples, our results demonstrate that UED can also be used to capture structural changes of solvated molecules. The direct access to interatomic distances shows that LUED can be used characterize the speed of dissociation, which could in the future be compared to gas-phase measurements and calculations to offer insight into how the solvent affects the potential energy surfaces governing dissociation.

The ability to directly resolve structural changes of a solute on femtosecond and few-picosecond time scales establishes LUED as a promising tool for characterizing chemical reaction dynamics in liquids. The large *Q* range available, and the small scale of the LUED instrument relative to x-ray free electron lasers, place LUED in a complementary position to existing XSS techniques. However, the high concentration and laser power necessary to obtain a signal even for the strongly scattering $I_{3}^{-}$ system point to limitations in the current applicability of the LUED technique to lighter and more complex systems in solution. The maximum difference signal magnitude relative to the liquid peak in this experiment was only 0.4% in the *Q* range that could be interpreted by independent atom model calculations; in addition, more than 50% of this signal arises from solvent heating. Although the difference scattering approach automatically subtracts out the solvent background, any fluctuation due to sample or beam instabilities can make observing the desired per-mil signals challenging. Studies of neat solvent, with percent-level difference signals, have already proved possible (Ref. 45, submitted). Planned improvements to the LUED setup will help address these issues, including a near-term upgrade of the repetition rate by a factor of three, from 360 to 1080 Hz, that will decrease data collection times.^9^ Additionally, replacement of the integrating-mode detector with a direct electron detector with single-electron and shot-by-shot capabilities^46^ will allow for more sophisticated data treatment, including shot-to-shot differencing, a technique which has been shown to improve signal-to-noise in transmission-mode x-ray studies.^47,48^ Further development of the liquid jet system, including more stable gas flow and improved nozzles, could improve jet stability and therefore data quality. The combination of these improvements should deliver significant improvement in signal-to-noise in the next few years.

## METHODS

### Experimental setup

Optical laser pump/MeV electron probe experiments were carried out at the SLAC MeV-UED instrument in the liquid phase endstation described in Ref. 9. The sample was prepared by combining 130 mM I_2_ and excess KI salt (500 mM) in aqueous solution, with pH adjusted to 4 with perchloric acid. The equilibrium constant for the formation of $I_{3}^{-}$ from I_2_ and I^−^ strongly favors $I_{3}^{-}$^49^ and the absorptivity of $I_{3}^{-}$ at 400 nm is 25 times greater than that of I_2_,^50^ so any photoexcitation of I_2_ would be negligible.

The sample was introduced into the vacuum chamber via a gas-accelerated liquid sheet jet with thickness below 200 nm.^8^ The sample flow rate was 0.25 ml/minute. The second harmonic (400 nm) of a Ti:sapphire laser with 360 Hz repetition rate was used to pump the sample, while the 3.7 MeV probe electrons were created from its third harmonic in an electron gun. Probe electrons scattered from the sample were collected on a phosphor screen 3.2 meters downstream. A delay stage was used to vary the arrival time of the optical pump relative to the electron probe. The overall time resolution of the experiment is determined by the temporal bunch length of the electrons. In a low electron bunch charge mode (∼2 fC/pulse), ultrafast temporal resolution is achieved and is estimated to be 180 fs full width half maximum (FWHM).^9^ In a high bunch charge mode (∼100 fC/pulse), picosecond temporal resolution is achieved and is typically between 0.5 and 1 ps FWHM. The pump laser spot size was 160 × 270 *μ*m FWHM; pulse energy for high bunch charge data was 40 *μ*J (120 mJ/cm^2^), while for low bunch charge data, the laser pulse energy was 60 *μ*J (180 mJ/cm^2^). Optical transient absorption measurements at 180 mJ/cm^2^ were carried out to quantify the effect of this high laser fluence, as described in supplementary material Sec. II.

Scattering patterns were integrated for 5 s (1800 shots) per image. In a single scan of pump-probe time delay, 5 images were taken at each delay, with the order of the delays randomized during each scan to decrease systematic errors. The high bunch charge dataset consists of 135 scans while the low bunch charge dataset consists of 118 scans. The images were background-subtracted and filtered as described below. The processed images were averaged and the pixels radially binned by distance from the beam center, resulting in a one-dimensional curve as a function of momentum transfer Q=(4π/λ) sin (θ/2), where *λ* is the de Brogie wavelength of the 3.7 MeV electrons (0.3 pm) and *θ* is the scattering angle. Difference scattering was calculated by subtracting an average curve measured at negative time delays (electrons arrived first) from the entire dataset. No anisotropy was observed in the difference scattering prior to radial binning, likely due to an insufficient signal-to-noise ratio.

The temperature of the sample was estimated by comparison of the total scattering pattern (which mostly encodes the structure of water) to patterns obtained from molecular dynamics simulations of water at various temperatures, as shown in Fig. S2. The temperature is estimated to be 340 K in the low bunch charge data and 330 K in the high bunch charge data. The temperature of the sample is significantly above room temperature due to heating of the liquid delivery nozzle by the closely positioned heated sample catcher.^9^ The variation in temperature between datasets results from slight differences in the alignment of the nozzle.

### Data treatment

Electron scattering patterns were collected via a phosphor screen and mirror assembly projecting the image onto an Andor iXon Ultra 888 camera.^9^ A “dark” background was removed by subtracting the mean value of pixels at the corner of the detector, outside of the circular image of the phosphor screen. Pixels inside or close to the central hole in the phosphor screen (which allows the main electron beam to pass through) were masked. The images were treated to remove saturated pixels or rows of pixels due to stray x-ray photons on the detector. The position of the electron beam was fit by selecting all pixels within a narrow range of signal values and fitting a circle to these points. This was done for three signal levels on each image, and the average center of the three circles used as the per-image beam center. The center-finding algorithm was also performed on an average image composed of all images in a given data run. Individual images where the beam center was more than two standard deviations from the average in either *x* or *y* were removed from the analysis. Images were normalized to the total counts within a donut-shaped mask excluding the main beam and edges of the detector.

These normalized images were then binned by pump-probe time delay and averaged. One-dimensional curves as a function of distance from the center were formed by binning pixels by their distance from the average-image center point. This distance was calibrated into *Q*-space using the small angle approximation by multiplying by a scaling constant determined by a reference experiment on a solid Au sample. Difference scattering was then obtained by subtracting averaged negative pump-probe delays from the whole dataset. In the low bunch charge data, a linear tilt of the difference scattering was also apparent, possibly due to slight fluctuations in the thickness of the liquid jet. Increased thickness would cause increased multiple scattering, moving intensity from the elastic peaks to a broader distribution with more intensity in high *Q* values. This would lead to a randomly directed tilt in the difference data, which was observed. This tilt was removed by subtracting a line fit to the difference data at each time point, forcing the difference scattering to center around zero. This tilt removal changed the position of the peaks shown in Fig. 4 by less than 0.5%.

### MD simulations

MD simulations were carried out using the Desmond software package developed at D. E. Shaw Research.^51^ Geometries and partial charges on the iodines were taken from Ref. 15 (and shown in supplementary material Table S1), while the Lennard–Jones parameters were taken from the OPLS 2005 force field. The geometries of the bound iodines were kept fixed by applying harmonic positional restraints with a force constant of 500 kcal/mol, and solvated in a cubic box with 35 Å side length containing water molecules. The TIP4P-Ew force field was used for water. The simulations were performed in the NVT ensemble using a Nose–Hoover thermostat at 300 K, with a time step of 2 fs for 2 ns, collecting frames every 1 ps. Therefore, approximately 2000 frames were used for the calculation of the radial distribution functions (RDFs), which were sampled in 0.01 Å. Example solute–solvent RDFs are shown in supplementary material Fig. S5. MD simulations were also performed at a temperature of 330 K (the temperature of the experiment). The difference scattering signals obtained from these simulations differed from the 300 K signals by less than 0.5% and were not further considered.

## SUPPLEMENTARY MATERIAL

See the supplementary material for additional figures and details of the experiment and analysis.

## Data Availability

The data that support the findings of this study are available from the corresponding author upon reasonable request.
